# Streamline Flow of the Portal Vein Affects the Distribution of Colorectal Cancer Metastases: Clinical Reality or Just a Belief? A Systematic Review and Meta-Analysis

**DOI:** 10.3390/cancers16233902

**Published:** 2024-11-21

**Authors:** Stavros Savvakis, Vasileios I. Lagopoulos, Stylianos Mantalovas, Eleni Paschou, Periklis Kopsidas, Christina Sevva, Alexandros Vasileios Karakousis, Eleni Gigi, Isaak Kesisoglou

**Affiliations:** 1 3rd Surgical Department, AHEPA University Hospital of Thessaloniki, School of Medicine, Aristotle University of Thessaloniki, 1st St. Kiriakidi Street, 54621 Thessaloniki, Greece; savvstav@auth.gr (S.S.); lagopoulo@auth.gr (V.I.L.); smantalov@auth.gr (S.M.); pkopsidas@auth.gr (P.K.); chrissevva@auth.gr (C.S.); vkarakob@auth.gr (A.V.K.); ikesis@auth.gr (I.K.); 2 2nd Internal Medicine Department, Aristotle University of Thessaloniki, Hippokrateio General Hospital, 54642 Thessaloniki, Greece; elengigi@auth.gr

**Keywords:** colon cancer, liver, metastasis, portal vein, turbulent, blood flow

## Abstract

**Background:** The “streamline flow” of the portal vein (PV) is a controversial yet well-known theory presented almost 125 years ago. It states that blood from the superior mesenteric vein (SMV) goes preferentially to the right liver lobe, while splenic and inferior mesenteric veins divert blood predominantly to the left lobe, affecting this way the metastatic distribution of colorectal cancer (CRC). The aim of this systematic review is to assess its validity by examining and combining all the relevant literature with a critical eye. **Materials and methods:** This study constitutes a systematic review of the literature and adheres to all PRISMA system criteria. Three search engines (PubMed, Google Scholar, and the Cochrane Library) were used, and 11 out of 435 studies between 1984 and 2024 were finally included in this meta-analysis. All statistical analysis was conducted using RevMan Web, Version: 7.12.0, and Jamovi v 2.3.260 software. **Results:** The meta-analysis revealed that regardless of the primary location of the tumor, the metastases preferentially migrate to the right lobe due to various possible reasons. The prevalence of metastases from right colon cancer to the right liver lobe was 75%, while the prevalence of left colon cancer metastases to the right lobe was 68%. This difference was not found statistically significant. **Conclusions:** The theory has been proven inaccurate, at least from the perspective of the origin-associated distribution of CRC metastases in the liver lobes, and therefore bears no clear predictive value. Further research under different perspectives is essential for determining more definite conclusions.

## Simple Summary

Contrary to the traditional theory of streamline flow in the portal vein, our meta-analysis showed that regardless of the primary location of the tumor, metastases preferentially migrate to the right lobe of the liver. This can be attributed, according to recent breakthroughs in blood hydrodynamics, to the blood flow being physiologically turbulent, the Taylor’s dispersion phenomenon, or to the complicated role of circulating tumor cells, as well as the more well-established difference in the volume and mass ratios among the two lobes, the difference of blood flow per minute between them, and various anatomical reasons.

## 1. Introduction

CRC is recognized as the third most prevalent cancer and the second leading cause of cancer-associated mortality globally, while in the last two decades a concerning increase in its incidence among younger patients (under the age of 50) has been noticed [1,2]. Initial assessment reveals metastatic disease in approximately 15% to 30% of patients, while a significant proportion, estimated between 20% and 50%, presents metastatic lesions in later stages, despite the initial diagnosis of localized disease. The liver emerges as the primary site of metastatic spread, facilitated mostly by the PV, which, as we know, drains blood from the abdominal viscera and obviously acts as an “escape highway” for tumor cells of colorectal origin. The PV is formed by the junction of the SMV and splenic vein (SV) beside the neck of the pancreas, and after a common trunk of 7–10 cm divides into the right and left branches that enter the liver parenchyma. The SMV typically drains blood from the small bowel and the so-called right colon, i.e., the cecum, the ascending and right 2/3 of the transverse colon [3,4,5]. The SV, just before the junction with the SMV and posteriorly to the tail of the pancreas, accepts venous supply from the left colon (left 1/3 of the transverse, descending colon, sigmoid, and upper rectum) through the inferior mesenteric vein (IMV) [6].

This binary blood flow pattern to the liver gained attention by anatomists in the early 20th century, prompting investigations initially in animals. In 1901, Serege conducted experiments by injecting India ink into the PV tributaries of dogs. He observed a preferential distribution of blood flow, with the one supplied from the SMV heading mainly to the right branch of the PV, and the one coming from the SV reaching mostly the left branch of the PV. [7] This led to the introduction of the PV “streamline flow” theory, suggesting that the relatively short trunk of the PV does not allow the bloodstream of the SMV and the SV (IMV) to be mixed adequately, and therefore the right and left branches of the PV carry blood mostly from the SMV and the SV, respectively. [8] Further animal studies offered support for this theory; however, some researchers presented findings that rejected this hypothesis. [9] The ambiguity of results on this topic has persisted over the years, even in studies conducted on human subjects, and despite the considerable advancement in imaging technology, conclusive outcomes have not been obtained.

We performed a systematic review of all published studies on the streamline flow phenomenon in human patients with liver metastases from CRC. The aim of our review was to assess the validity of this specific theory and, if confirmed, explore its potential clinical significance.

## 2. Materials and Methods

### 2.1. Search Strategy

The present systematic review adhered to the guidelines set forth by the Preferred Reporting Items for Systematic Reviews and Meta-analysis (PRISMA) statement and followed the recommendations of the Cochrane Collaboration. We identified original articles focusing on the “streamline flow” of CRC metastases to the liver lobes. No restrictions concerning article language were implemented. The search utilized the following free-text terms in the Medline, Google Scholar, and Cochrane Library databases: (‘colorectal metastases’) AND (‘streamline flow’) AND (‘portal vein’) OR (‘hepatic lobes’) AND (‘distribution’). Given that this study constituted a systematic review of the published literature, neither informed consent nor ethical clearance was deemed requisite. Subsequently, the study was registered in the PROSPERO database (Registration ID CRD42024562722).

### 2.2. Study Selection

A thorough electronic search of Medline, Google Scholar, and the Cochrane Library databases was conducted between 10 December 2023 and 10 January 2024 to identify studies concerning the research query. There were no constraints regarding the publication year of the identified studies. To ensure maximum sensitivity, comprehensive manual searches and evaluations were conducted on the “References” sections of all relevant review articles and full-text articles selected for inclusion.

Two authors (V.I.L. and S.S.) conducted a comprehensive literature search independently. In instances where there was a discrepancy between the assessments of the manuscripts by the two reviewers, a third reviewer (S.M.) was consulted to resolve the disagreement. Both the inclusion and exclusion criteria were predetermined before the initiation of the literature search. Only studies meeting the following criteria were eligible for inclusion: (1) surgical, radiographic, or autopsy studies; (2) liver metastases spread solely from cancer of the colon and the upper one-third of the rectum; (3) division of the liver into right and left lobes by Cantlie’s line; (4) patients of all ages and racial or ethnic backgrounds. Exclusion criteria consisted of: (1) animal studies; (2) studies lacking data that could be extracted; (3) case reports.

All the retrieved original articles were independently evaluated for eligibility by two authors (S.S. and V.I.L.), adhering to the aforementioned criteria for inclusion or exclusion. Subsequently, the qualifying studies were extracted. A data extraction sheet was employed by both authors to gather information from each eligible study, including demographic characteristics, primary outcomes, study design, and year of publication. The distribution of liver metastases in relation to the primary CRC location was the primary outcome.

### 2.3. Statistical Analysis

In this meta-analysis, we investigated the association between primary tumor location (right or left colon) and liver metastasis location (right or left liver as divided by Cantlie’s line) in patients with CRC. Data were extracted from studies reporting on metastasis distribution and categorized into the four following groups: right colon cancer with right liver metastasis, right colon cancer with left liver metastasis, left colon cancer with right liver metastasis, and left colon cancer with left liver metastasis. Risk ratios (RRs) were used to estimate the aggregated effect size, given the binary outcomes studied, and the prospective nature of the data recording as well as the inclusion of various study designs. RRs were plotted as forest plots. Heterogeneity was assessed using Cochran’s Q statistic and quantified by using I2 statistics. Articles were considered to have significant heterogeneity between studies when *p* < 0.1 or I2 > 50%, and the funnel plot was assessed for publication bias. A random-effects model was used to account for variability between studies. Statistical analysis was conducted using RevMan Web, Version: 7.12.0, and Jamovi v 2.3.260 software, and the results were considered significant at *p* < 0.05.

## 3. Results

**Figure 1:**
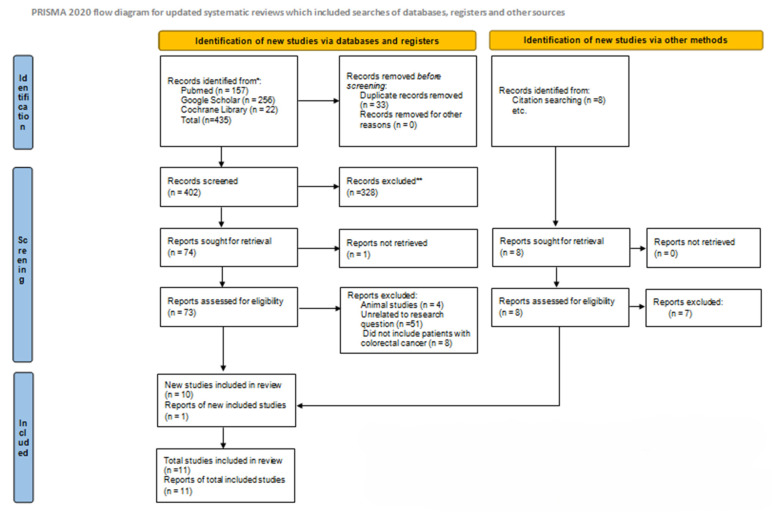
PRISMA flow diagram for study selection [20]. * Consider, if feasible to do so, reporting the number of records identified from each database or register searched (rather than the total number across all databases/registers). ** If automation tools were used, indicate how many records were excluded by a human and how many were excluded by automation tools.

Our initial search yielded 435 records. After accounting for duplicates, 33 of them were removed, and the remaining 402 articles were eligible for title and abstract screening. Consequently, 328 of them were rejected due to their lack of relevance, and the full text of the remaining 74 was assessed. One of these reports, however, could not be retrieved. From those 73 reports, 4 were excluded because they were animal studies, 51 were dismissed as unrelated to our research question, and 8 were excluded because they included patients with metastatic cancer other than colorectal. In addition to the remaining 10 articles, 8 articles were detected through the reference lists of the full text articles retrieved, and 1 of them was ultimately found eligible for inclusion. Therefore, our systematic review consisted of 11 studies in total (Figure 1) [6,10,11,12,13,14,15,16,17,18,19].

All studies were published between 1984 and 2024. The quantity of patients, and correspondingly, the number of metastatic colon cancers in each study, ranged from 7 to 891. Overall, a total of 2357 patients were included. The largest study was performed in 2014 by Pathak et al. [16] and it was a radiographic study involving 891 patients. Table 1 presents the main demographic characteristics of the included articles. Metastatic CRCs have been categorized into two groups: Right Colon and Left Colon cancers. The Right Colon pertains to the segment of the colon drained by the SMV, while the Left Colon refers to the part drained by the inferior mesenteric vein. Tumors located in the upper one-third of the rectum were placed within the same category as Left Colon cancers, on account of the drainage of this particular anatomical area by the inferior mesenteric vein.

The distribution of the metastases to the liver lobes was examined in 667 patients with primary cancer located to the Right Colon (Table 2a), and in 1690 patients with Left Colon tumor (Table 2b). A total of 855 liver metastases were derived from Right Colon cancers, with 75% identified in the right lobe and 25% in the left lobe of the liver. On the other hand, Left Colon malignancies disseminated 2479 liver metastases, of which 68% were directed to the right lobe and 32% to the left liver lobe.

**Figure 2:**
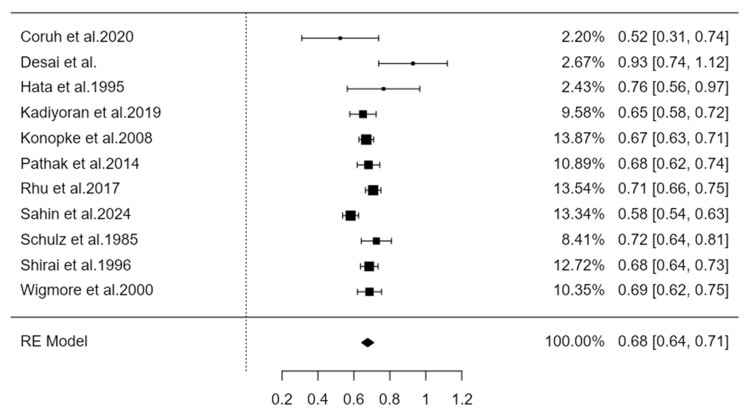
Forest plot representing the pooled incidence of metastases from the left colon to the right liver [6,10,11,12,13,14,15,16,17,18,19].

**Figure 3:**
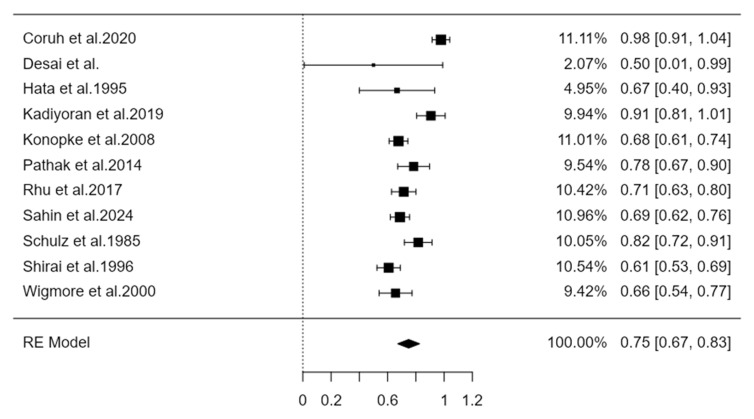
Forest plot representing the pooled incidence of metastases from the right colon to the right liver [6,10,11,12,13,14,15,16,17,18,19].

Similarly, the synthesis of findings on metastases from the right colon to the right liver, also based on random effects models, shows an estimated prevalence of 0.75 with a 95% confidence interval (0.67–0.83). The heterogeneity index I^2^ for this synthesis was estimated at 88.3%, a value considered high according to the literature (Figure 2 and Figure 3).

Close examination of these findings suggests that the confidence intervals for both approaches are completely overlapping, indicating no significant differences in metastases to the right liver, regardless of whether they originate from the right or left colon. A statistical comparison will be conducted to confirm this conclusion.

**Figure 4:**
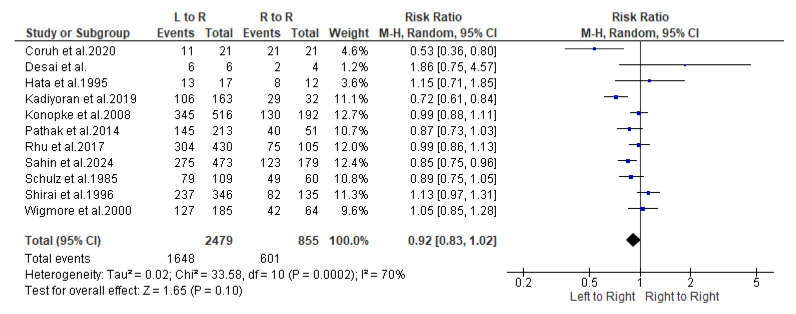
Forest plot representing the pooled incidence of metastasis rates from the “Left colon to right liver” and “Right colon to right liver”, based on random effects models using the Mantel–Haenszel variance method [6,10,11,12,13,14,15,16,17,18,19].

**Figure 5:**
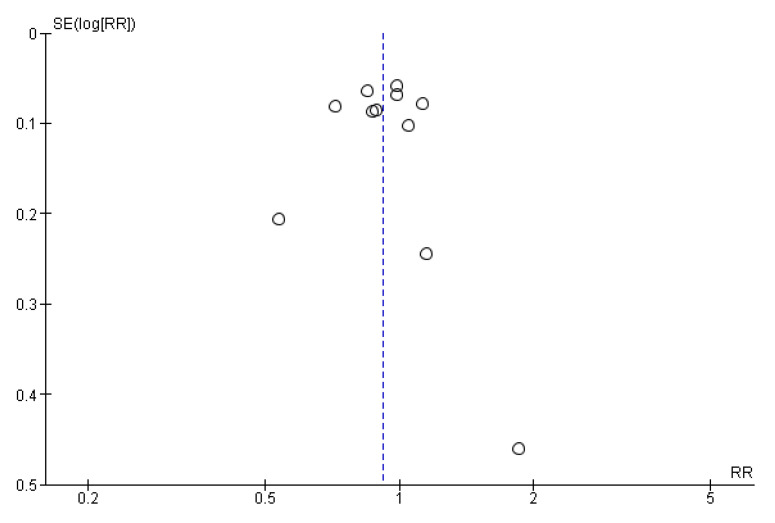
Publication bias assessment.

Following is the synthesis of study results regarding metastasis rates from the “Left colon to right liver” and “Right colon to right liver”. The estimation, based on random effects models using the Mantel–Haenszel variance method, yielded a heterogeneity index I^2^ of 70%, a value deemed quite high according to the literature. The aggregated estimate for the Risk Ratio (RR) was 0.92 with a 95% confidence interval of 0.83–1.02; *p* = 0.100, suggesting that the probability of metastases to the right liver does not significantly differ based on whether they originate from the right or left colon (Figure 4). The funnel plot indicated low levels of publication bias due to its symmetry (Figure 5).

**Figure 6:**
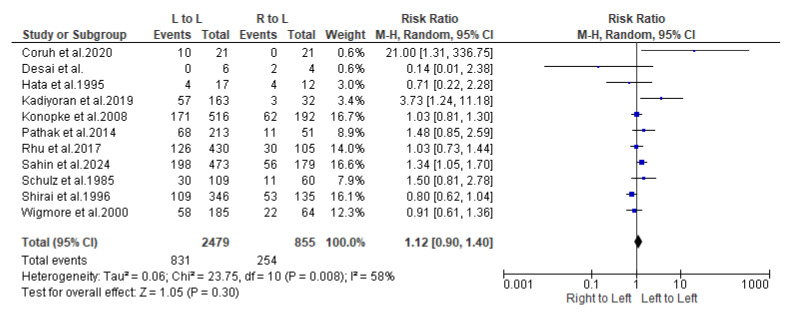
Forest plot representing the pooled incidence of metastasis rates from the “Left colon to left liver” and “Right colon to left liver”, based on random effects models using the Mantel–Haenszel variance method [6,10,11,12,13,14,15,16,17,18,19].

A subsequent sensitivity analysis examined the potential impact of excluding any of the included studies. The analysis demonstrated that even when studies with the highest weights were omitted, the overall conclusions remained unchanged. Consequently, the lack of statistical significance is consistent across all cases, indicating that the probability of metastasis from the “Right colon to left liver” and “Left colon to left liver” does not differ significantly (Figure 6).

## 4. Discussion

To the best of our knowledge, this constitutes the first systematic review and meta-analysis of published studies examining the theoretical streaming of CRC metastases to the liver lobes via the PV. Our objective was to provide conclusive answers to the specific research question originally raised by Serege in 1901. He injected India ink into the PV tributaries of dogs and observed that blood from the SMV diverted preferentially to the right liver lobe, while blood from the SV tended to direct preferentially to the left lobe [7]. In the same year, Glenard confirmed these findings. However, in 1909, Bauer et al. were the first to challenge the theory, describing a homogeneous distribution of India ink throughout the liver regardless of the portal tributary injected [21]. Many researchers who followed utilized a variety of methods and technologies to either support or invalidate the notion, leading to varying outcomes. The approach of this paper to the streamline flow hypothesis was more clinically orientated, as we chose to analyze studies of human patients with liver lobe metastases from CRC. The most recent study included, carried out by Sahin et al. [19], comes approximately 120 years after Serege’s initial proposal of the theory, underscoring its continued relevance and interest.

According to the pre-mentioned results of the study, the prevalence of metastases from right colon cancer heading to the right liver lobe was estimated at 75%. On the other hand, the prevalence of metastases from the left colon to the right liver lobe was estimated at 68%. The statistical comparison indicated that the probability of metastases to the right liver does not differ depending on whether it originated from the right or left colon. Regardless of the location of the primary tumor, the metastases preferentially migrated to the right lobe, and each lobe of the liver does not show a preference for any segment of the colon. The observed difference in the prevalence of metastases to the right lobe from the right colon (75%) and the left colon (68%), though not statistically significant, can be attributed to various factors.

It is well established by various authors that the ratio of weight and volume between the right and left lobes of the liver is approximately 2:1 [11,14,15,22]. Research involving 1000 living donors in Korea found that the right liver accounted for 65.3% of the total liver volume, while the left liver made up 34.7%, translating to a right-to-left volume ratio of 1.88:1 [23]. Some sources have even reported a more extreme ratio of 6:1 [24]. Nevertheless, the most common finding remains that the volume ratio between the right and left lobes of the liver is 2:1. Accordingly, assuming a homogeneous distribution of metastases via the PV, the ratio of metastases in the two lobes should also be 2:1. This seems to apply with the previously calculated prevalences: 75% (3/4) of the metastases derived from the right colon went to the right liver, and 25% (1/4) went to the left liver. Similarly, 68% (approximately 2/3) of the left colon cancer metastases directed to the right liver lobe and 32% (approximately 1/3) to the left liver lobe.

Another plausible explanation for the difference in prevalence mentioned above is the higher blood flow delivered to the right lobe by the right branch of the PV (RBPV), compared to the flow from the left branch (LBPV) to the left lobe. Kutlu et al. conducted a study using Quantitative Doppler evaluation on 30 healthy volunteers [25], revealing that the RBPV had an average blood flow rate of 666 mL/min with a standard deviation of ±168. In contrast, the LBPV had a mean flow rate of 445 mL/min with a standard deviation of ±174. The flow ratio RBPV/PV (portal vein) was found to be 0.6, while the LBPV/PV ratio was 0.4. By dividing these values, the ratio of the flows of the RBPV and the LBPV is found to be 1.5:1, which is consistent with the distribution of metastases observed in our meta-analysis.

The tendency of metastases to favor the right lobe might also be influenced by anatomical factors. Typically, the PV constitutes a straight upward continuation of the SMV, which drains blood from the right colon [19]. Therefore, a small, localized streamline phenomenon may occur, directing metastases from the right colon more readily to the right liver lobe. Another interesting hypothesis was made by Pathak et al. [16]. They compared the anatomical angulation of the RBPV with that of the right main bronchus, which is well known to be the most common and favorable route for aspirated foreign objects [26]. The RBPV is typically a continuation of the PV, while the LBPV branches off from the PV at an acute angle [18]. A similar mechanism may partially account for the difference in the prevalence of metastases to the right lobe from the right colon and the left colon.

Blood flow is modeled as streamline laminar flow in the vast majority of research, which involves a consistent blood flow within a vessel where each blood layer stays at an equal distance from the vessel wall based on the Womersley flow model (WFM) [27]. This phenomenon is also seen in nature beyond the circulatory system. In the Amazon basin or even in Sudan’s capital, Khartoum, the waters of two distinct rivers do not mix because of differences in their physical and chemical properties, creating an impressive two-colored streamline flow [28]. Nonetheless, the PV is a double Y or H-shaped vascular formation, and liquid physics obliges some turbulence on the site of bifurcation to the right and left branches. Consequently, there is a high probability of dispersion phenomena, and that does actually happen, according to Taylor and more recent researchers [29,30,31]. During a laminar flow in a T-shaped cylindrical pipe (in this case, the PV), there is a transverse diffusion zone of spherical particles (in this case, the metastases) located between the fluid streams. Botar et al. performed a computational fluid analysis and Echo-Doppler simulation and proved that these mathematical and chemical models also apply in the human PV, in vivo [32]. Taking the above into consideration, the dispersion of metastases may explain the statistically insignificant difference between the origin of colorectal malignancy and the liver lobe affected by the metastases.

The distribution of CRC metastases to the liver lobes becomes even more complicated, considering the role of circulating tumor cells (CTCs), which can be present in the bloodstream either as isolated CTCs or even in clusters of different sizes. CTCs derived from the primary site enter the blood circulation or lymphatic system in numerous ways and frequently unify with a plethora of blood cells, such as neutrophils, lymphocytes, macrophages, platelets, and other cells, forming clusters that can migrate to the liver or other distant organs [33,34,35]. The fact that they have been detected in hepatic biopsies in 10% of patients who underwent surgery due to CRC (stages I–III) [36], underlines their important role in the metastatic disease. This means that in order for the accuracy of vascular hemodynamic models to improve, analysis should be conducted to include cell dynamics and fluid–solid interaction with vessel walls [37].

**Figure 7:**
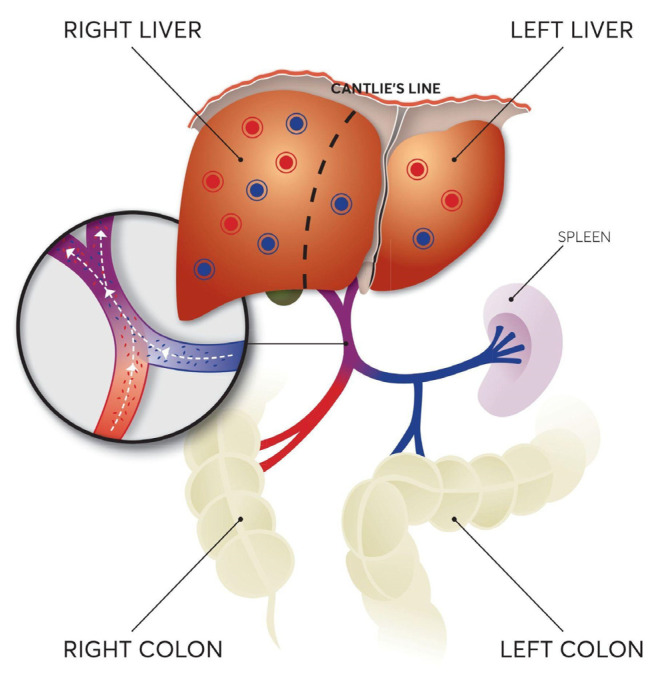
Blood Flow in the Portal Vein (PV). In this figure, blood and circulating tumor cells coming from the right colon are depicted as red, while those coming from the left colon are blue. Liver metastases arising from right colon cancer are colored red, while metastases from left colon cancer are colored blue. Metastases are depicted to preferentially migrate to the right lobe, which can be attributed to the difference in the volume and mass ratios among the two lobes and the difference of blood flow per minute between them, with blood in the PV being purple in the figure due to a degree of mixing due to physiologic turbulent blood flow in the PV, as newer data suggest.

Even though our study provides interesting insights, its limitations should be acknowledged. First of all, the number of studies that examine the PV flow at the microcirculatory level is limited. When assuming laminar flow in a system, there is still some degree of mixing on a microscale which occurs primarily through diffusion. The area where mixing happens is the diffusion zone (DZ), and it is located in the center of the vessel. The width of the DZ, which is usually noted as δ(x) in the literature, increases as the fluids flow along the vessel [38]. Thus, the degree of blood mixing should be investigated more thoroughly. Contemporary research has also supported that the physiologic blood flow is turbulent under physiologic circumstances (Figure 7), which means that blood flows in a multidirectional manner within the vessels, resulting in continuous mixing throughout the PV [27,37]. In reality, blood flow is pulsatile and multi-harmonic, while blood is a non-Newtonian fluid, meaning that the main body of existing literature is based on the simplified ideal approximation, the nonetheless pioneering WFM [37]. In light of this, newer research focused on developing more complex models that take into account the aforementioned properties and seems to conclude that blood flow is actually, at least to some degree, physiologically turbulent despite the relatively low Reynolds number of blood [39]. Saqr et al. showed that physiologic blood flow is sensitive to initial conditions, meaning it is subjected to the properties of chaos theory; it displays global hydrodynamic instability and undergoes a kinetic energy cascade of non-Kolmogorov type [37]. This kind of exception has also been demonstrated to apply in atmospheric turbulence [40]. Furthermore, Yambe et al. also demonstrated the possible existence of lower-dimensional chaotic dynamics in blood flow [41].

If true, these theories render the blood flow in the PV and the distribution of metastases to the hepatic lobes an even more complicated phenomenon that undoubtedly necessitates further research on turbulent flow. It should also be highlighted that we examined the streamline flow phenomenon only from one perspective, based on the distribution of metastases from the right and left colon to the liver lobes. Nevertheless, other liver pathologies such as cystic echinococcosis [42], amebic liver abscesses [43], or even pyelophlebitis [44] may be influenced by the flow in the PV; thus, conducting meta-analyses on these conditions is likely to give a more clear view and understanding of the streamline flow phenomenon in the PV.

Lastly, the clinical significance of the streamline flow phenomenon is probably limited for surgeons, as decisions regarding liver metastasis resection are primarily based on factors such as the number of metastases, the Milan criteria, the patient’s performance status, and the volume of the remaining liver parenchyma [45]. However, the anatomical and scientific significance of this phenomenon is considerable, giving valuable insights into the intriguing anatomy and complex physiology of the human body. Moreover, a physiologically accurate model of blood flow in the PV system could have significant impact in interventional radiologists’ clinical practice. Such modeling might enable more targeted perioperative interventions, such as eclectic perioperative embolization, as an alternative to traditional PV embolization, optimizing liver hypertrophy while minimizing the risk of subsequent tumor volume increase, potentially improving treatment outcomes for patients [46]. However, further research is needed in order to explore these possibilities.

## 5. Conclusions

According to the traditional theory of streamline flow in the PV, metastases from the right colon go preferentially to the right liver lobe (through SMV), while metastases from the left colon prefer to direct to the left liver lobe (through the inferior mesenteric vein). On the contrary, our meta-analysis showed that regardless of the primary location of the tumor, the metastases preferentially migrate to the right lobe, which can be attributed to the different volume and mass ratios among the two lobes, to the different blood flows per minute and blood flow being physiologically turbulent, various anatomical reasons, to the Taylor’s dispersion phenomenon, or even to the complicated role of CTCs. The statistical analysis also concluded that each lobe of the liver does not exhibit a preference for any segment of the colon. The observed difference in the prevalence of metastases to the right lobe from the right colon (75%), and the left colon (68%), though not statistically significant, could be explained by a mini-streamline flow at the microcirculatory level, favored by the anatomical angles of the converging vessels. This is the first meta-analysis that offers a high quality of evidence in a theory concerning many researchers for more than 120 years.

## Figures and Tables

**Table 1:** Demographic characteristics of the included studies [6,10,11,12,13,14,15,16,17,18,19].

Author	Year	Country	Study Design	Type	n	Population	Gender (Male/Female)
Desai et al.	1984	USA	N/R	Radiographic	40	Adults	N/R
Schulz et al.	1985	Germany	Retrospective	Autopsy study	7	N/R	N/R
Hata et al.	1995	Japan	Retrospective	Surgical & Radiographic	29	N/R	N/R
Shirai et al.	1996	Japan	N/R	Surgical	85	Adults	52/33
Wigmore et al.	2000	UK	Prospective	Radiographic	207	Adults	137/70
Konopke et al.	2008	Germany	Prospective	Surgical	178	Adults	109/69
Pathak et al.	2014	UK	Prospective	Radiographic	891	N/R	N/R
Rhu et al.	2017	Korea	Retrospective	Surgical	410	Adults	245/165
Kadiyoran et al.	2019	Turkey	Retrospective	Radiographic	326	Adults	221/115
Coruh et al.	2020	Turkey	Retrospective	Radiographic	86	Adults	63/23
Sahin et al.	2024	Turkey	Retrospective	Radiographic	96	Adults	63/33

n: number of patients, Ν/R: not reported.

**Table 2:** (**a**) Distribution of Right Colon Metastases to the Liver. (**b**) Distribution of Left Colon Metastases to the Liver [6,10,11,12,13,14,15,16,17,18,19].

(a)				
Study	N	n	Right Liver (n)	Left Liver (n)
Desai et al.	21	21	100 (21)	0 (0)
Schulz et al.	3	4	50 (2)	50 (2)
Hata et al.	12	12	66.6 (8)	33.3 (4)
Shirai et al.	18	32	90.6 (29)	9.4 (3)
Wigmore et al.	51	192	67.7 (130)	32.3 (62)
Konopke et al.	35	51	78.4 (40)	21.6 (11)
Pathak et al.	169	105	71.4 (75)	28.6 (30)
Rhu et al.	121	179	69 (123)	31 (56)
Kadiyoran et al.	196	60	81.6 (49)	18.4 (11)
Coruh et al.	22	135	61 (82)	39 (53)
Sahin et al.	19	64	65.6 (42)	34.4 (22)
(b)				
Study	N	n	Right Liver (n)	Left Liver (n)
Desai et al.	19	21	52.3 (11)	47.6 (10)
Schulz et al.	4	6	100 (6)	0 (0)
Hata et al.	17	17	76.5 (13)	23.5 (4)
Shirai et al.	67	163	65 (106)	35 (57)
Wigmore et al.	156	516	66.9 (345)	33.1 (171)
Konopke et al.	143	213	68 (145)	32 (68)
Pathak et al.	722	430	70.6 (304)	29.4 (126)
Rhu et al.	289	473	58 (275)	42 (198)
Kadiyoran et al.	130	109	72.5 (79)	27.5 (30)
Coruh et al.	64	346	68.5 (237)	31.5 (109)
Sahin et al.	77	185	68.7 (127)	31.3 (58)

N: number of Colon Cancers; n: number of liver metastases.

## Data Availability

All data used in this meta-analysis is available from the corresponding author and can be provided upon reasonable request.
